# The millipede family Ammodesmidae (Diplopoda, Polydesmida) in western Africa

**DOI:** 10.3897/zookeys.221.3739

**Published:** 2012-09-11

**Authors:** Didier VandenSpiegel, Sergei Golovatch

**Affiliations:** 1 Royal Museum for Central Africa, Invertebrate Section, B-3080 Tervuren, Belgium; 2 Institute for Problems of Ecology and Evolution, Russian Academy of Sciences, Leninsky pr. 33, Moscow 119071 Russia

**Keywords:** Diplopoda, Polydesmida, Ammodesmidae, *Ammodesmus*, taxonomy, new species, new synonymy, western Africa

## Abstract

Ammodesmidae are represented in western Africa by two species of a single genus, *Ammodesmus* Cook, 1896 (= *Cenchrodesmus* Cook, 1896, syn. n.). The type-species *Ammodesmus granum* Cook, 1896 (= *Cenchrodesmus volutus* Cook, 1896, **syn. n.**) is redescribed, based on neotype selection, as well as on additional samples, often containing numerous specimens, from Liberia, Guinea and the Ivory Coast. A new species is described from Mount Nimba, Guinea: *Ammodesmus nimba* **sp. n.**

## Introduction

The small Afrotropical family Ammodesmidae has hitherto been known to comprise only three genera. One of these is *Elassystremma* Hoffman & Howell, 1981, a recently reviewed oligotypic genus currently comprising four species from Kenya, Tanzania and Malawi (Hoffman and Howell 1981, VandenSpiegel and Golovatch 2004). Two further genera, both monobasic and both described from western Africa, have remained enigmatic ever since their proposals.

The taxonomic history of the family Ammodesmidae is rather confusing (Jeekel 1970). It was originally introduced invalidly, because it contained two genera, *Ammodesmus*, a nomen nudum, and *Doratodesmus* Cook, 1895, the latter name proposed to replace the preoccupied *Doratonotus* Pocock, 1894 (Cook 1895). Cook (1896a) validated Ammodesmidae only through selecting and diagnosing *Ammodesmus granum* Cook, 1896 as type species. A little later, Cook (1896b) slightly rediagnosed *Ammodesmus* and also described the new genus *Cenchrodesmus* for the sole species *Cenchrodesmus volutus* Cook, 1896, he had collected syntopically in Liberia together with *Ammodesmus granum*. This latter species was said to have been based upon the holotype while *Cenchrodesmus volutus* upon three syntypes. Both were first mentioned to have been taken from “the western part of the country”, but the exact locality, Mt Coffee, was soon provided elsewhere (Cook 1896c).

The diagnoses of the two genera and species were rather anecdotal and provided little useful information (Cook 1896b, page 414): “*Both genera have the habit of coiling into a sphere. The second segment is enormously enlarged so as to completely conceal the head and the first segment when viewed from the side as well as to cover the space left between the decurved carinae of the other segments when the creatures are coiled*. Ammodesmus *has the dorsum roughened by a transverse row of large papilliform tubercles rising from the posterior part of each segment, while* Cenchrodesmus *has the segments nearly smooth. When disturbed it coils up and lies motionless, and then is perfectly concealed, having exactly the appearance of a grain of sand*”. In summary, Cook (1895, 1896a-c) distinguished the family Ammodesmidae, as well as both *Ammodesmus* and *Cenchrodesmus*, by their extremely small size, claimed to be the smallest in Polydesmida (only about 2 mm in length), and their ability to volvate.

As no genital structures had been mentioned, VandenSpiegel and Golovatch (2004) suggested that all type specimens from Liberia might have been females. Furthermore, since the types could not be relocated for revision, *Ammodesmus* and *Cenchrodesmus* have ever since remained “*nomina dubia*” (Hoffman 1980). So even when Hoffman and Howell (1981) described the new genus *Elassystremma* and its sole, and type, species *Elassystremma pongwe* Hoffman & Howell, 1981 from Tanzania, the need was again emphasized in revising the two western African genera before unequivocally assigning *Elassystremma pongwe* to the family Ammodesmidae. The same uncertain situation has also remained after the latest review of *Elassystremma* which added three further congeners from eastern tropical Africa (VandenSpiegel and Golovatch 2004).

Between 2008 and 2011, a rich material of diplopods was taken or amassed by the first author from Liberia, Guinea and the Ivory Coast. This large collection appears to contain a proportion of Ammodesmidae, fortunately also with males from each locality becoming available for study. Three different morphotypes could be distinguished at once, including the two forms described by Cook (1896a-c), as well as a new one with a very peculiar colour pattern. Moreover, quite unexpectedly, both of Cook’s species happen to be the most common and widespread, in larger samples always coexisting, with all specimens showing the papilliform metatergal tubercles typical of *Ammodesmus granum* being males and the samples with nearly smooth tergites representing juveniles or females, i.e. just like the situation described for *Cenchrodesmus volutus*. This striking sexual dimorphism (which somewhat resembles that observed in some volvatory species of *Eutrichodesmus* Silvestri, 1910, a genus of the Australasian family Haplodesmidae - see Golovatch et al. 2009a, b, 2010) allows for the following unequivocal identifications and synonymy to be proposed: *Ammodesmus granum* Cook, 1896 must have been based on a male holotype, while *Cenchrodesmus volutus* Cook, 1896 is its junior subjective synonym which seems to have been based on three female syntypes. The third form appears to be a new species of *Ammodesmus*, taken from a single locality (Mt Nimba) and described below.

The present paper provides a review of *Ammodesmus*, the sole ammodesmid genus that appears to populate western Africa. Its gonopod and numerous other characters are documented here for the first time, and compared to those of *Elassystremma*, the sole eastern Afrotropical counterpart ammodesmid. The type species *Ammodesmus granum* is redescribed, based on neotype designation, and a new species is added to this genus. A distribution map of and a key to the *Ammodesmus* species are also given.

## Material and methods

The bulk of material belongs to the collection of the Royal Museum for Central Africa (MRAC), Tervuren, Belgium, with only a few duplicates shared with the collections of the Zoological Museum, State University of Moscow (ZMUM), Russia and the Muséum national d’Histoire naturelle, Paris (MNHN), France, as indicated hereafter. All samples are stored in 70% ethanol. Photographs were made with a Leica digital camera Leica DFC 500 mounted on a Leica MZ16A stereomicroscope. Images were processed with a Leica Application Suite program. Specimens for scanning electron microscopy (SEM) were air-dried, mounted on aluminium stubs, coated with gold and studied in a JEOL JSM-6480LV scanning electron microscope.

## Taxonomy

### Family Ammodesmidae

http://species-id.net/wiki/Ammodesmidae

#### Emended diagnosis.

An oligotypic family of minute polydesmidans (1.4–5.0 mm long) with 18 or 19 body segments in both sexes, capable of rolling into a tight sphere. Conglobation pattern becoming typical from paratergum 4 onwards (Golovatch 2003). Animals usually easily recognizable by having paraterga 2 strongly enlarged, all postcollum metaterga being clothed with a cerategument (= cuticular secretion layer) and various tuberculations (sometimes better developed in the male than in the female). Head broader than long. Antennae short, strongly clavate; antennomere 5 longest and largest, distinctly enlarged, about as high as long. Collum small, at best only slightly covering the head from above, only moderately convex. Generally, dorsum highly convex; paraterga very strongly declined ventrad, often deeply incised caudally at base; ventral edge rounded, either not or extremely poorly lobulated, well projecting below venter/coxae. Telson small, fully exposed in dorsal view. Ozopore formula nearly normal, only slightly varying from 5, 7, 9, 10, 12, 13, 15–17(18) (*Ammodesmus*) to 5, 7, 9, 12, 15–17(18) (*Elassystremma*); ozopores opening flush on tergal surface at about anterior third of paraterga, this opening sometimes being concealed by preceding paratergum.

Sterna very narrow, coxae usually subcontiguous medially. Last male legs either modified (*Ammodesmus*) or not (*Elassystremma*). Gonopod aperture rather modest in size, transversely oval, not reaching sides of metazona ventrally.

Gonopods mostly complex; coxae globose, usually but not always strongly enlarged and deeply excavate in the middle (= gonocoel), cannulae very evident. Telopodites basically unipartite, slender or stout, sometimes with a small lateroparabasal outgrowth, only seldom strongly exposed (*Ammodesmus granum*), more usually deeply sunken into gonocoel, leaving only tips exposed. Seminal groove mostly running on mesal face, turning laterad due to telopodite twisting only distally to subapically, with a very evident (*Ammodesmus granum*) or small solenomere either devoid of or supplied with a hairy pulvillus; accessory seminal chamber absent.

#### Distribution.

Liberia, Guinea and Ivory Coast (western Africa), as well as Tanzania, Kenya and Malawi (the African eastern Arc Mountains).

#### Key to recognized genera of Ammodesmidae

**Table:** 

1	Last pair of male legs not modified. Eastern tropical Africa	*Elassystremma*
–	Last pair of male legs strongly modified (Fig. 23). Western tropical Africa	*Ammodesmus*

### Genus Ammodesmus

Cook 1896

http://species-id.net/wiki/Ammodesmus

Ammodesmus Cook, 1896
Cenchrodesmus Cook, 1896 syn. n.

#### Type species.

*Ammodesmus granum* Cook, 1896

#### Diagnosis.

(after Cook 1896b, with modifications) Minute polydesmidans (length 1.4–2.0 mm) with 18 or 19 body segments (16 or 17+1+T), or rings, in both sexes. Head small, epicranium and interantennal region finely and densely granulose, lower half setose. Three labral teeth equal in size and length. Antennae short; antennomere 5 longest and largest, strongly enlarged, about as high as long; antennomeres 5 and 6 each with a distodorsal group of 20 to 30 bacilliform sensilla; antennomeres 4, 5 and 6 each with a single macroseta on dorsal side near apical third; terminal antennomere with usual four apical cones. Collum rather large and moderately convex, nearly not covering the head from above, surface finely and densely granulose. Tergum 2 with particularly strongly enlarged, spatuliform paraterga, latter of following segments not enlarged; lateral end subtruncate, but rounded; overlap pattern typical from paratergum 4 onward. Ozopore formula: 5, 7, 9, 10, 12, 13, 15–17(18); ozopores opening flush on tergal surface about anterior third of paraterga, the opening sometimes being concealed by preceding paratergum. Limbus smooth. Telson relatively small, its posterior edge with a row of macrosetae, ventrolaterally with 2 macrosetae borne on small knobs; epiproct very short and stout, surmounted by four conspicuous macrosetae in pits (= apparently, a spinning apparatus); hypoproct subtrapeziform with a paramedian pair of macrosetae borne on small knobs. Sterna very narrow, most coxae subcontiguous medially. Legs moderately robust, rather short and setose. First ♂ tarsus with modified setae, each last ♂ tibia showing an elongated process bearing a long apical seta, tarsus reduced, claw vestigial; other legs not modified.

#### Distribution.

Western Africa (Liberia, Guinea and Ivory Coast).

#### Species included.

*Ammodesmus granum* and *Ammodesmus nimba* sp. n.

#### Key to *Ammodesmus* species

**Table:** 

1	Coloration pinkish to brownish with darker metaterga (Fig. 1). Male with metatergal tubercles; gonopod with a small coxa, leaving most of telopodite exposed	*Ammodesmus granum*
2	Colour pattern of metaterga spotty (Fig. 28). Male devoid of metatergal tubercles; gonopods with strongly enlarged coxae supplied with a prominent gonocoel	*Ammodesmus nimba* sp. n.

### Ammodesmus granum

Cook, 1896

http://species-id.net/wiki/Ammodesmus_granum

**Figures 1–6.**
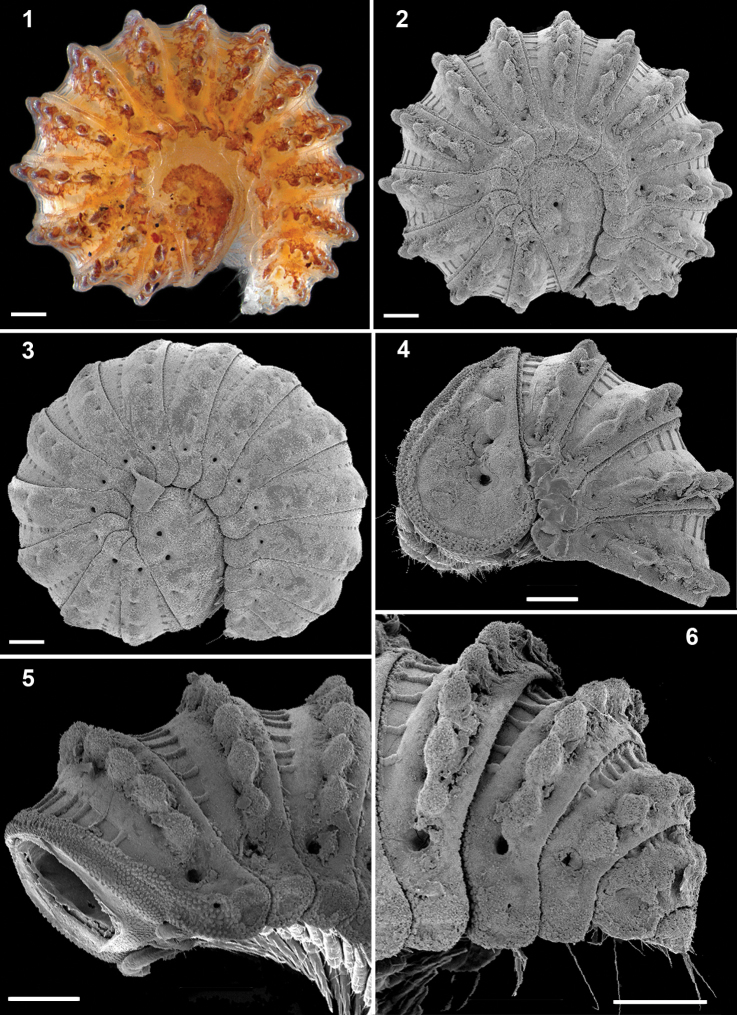
*Ammodesmus granum***1, 2** habitus of male, lateral view **3** habitus of female, lateral view **4–6** male, anterior, middle and caudal parts of body, respectively, lateral view. Scale bars: 100 µm.

Figs 1 [Fig F2] [Fig F3] –27

Ammodesmus granum Cook, 1896
Cenchrodesmus volutus Cook, 1896 syn. n.

#### Type material.

Neotype ♂ (MRAC 21667), Liberia, Bong Range Forest, 06°49'N, 010°17'W, rainforest, pitfall trapping, 13.III.2005, leg. D. Flomo.

This male specimen has been chosen as neotype, because it is in perfect condition and represents a near-topotype. A neotype of *Cenchrodesmus volutus* has also been selected to substantiate this taxon as well.

#### Other material.

1 ♂, 2 ♀ (ZMUM), 1 ♂ (MNHN), same locality, date and collector; 6 ex. (MRAC 21966) GUINEA, Mt Nimba, Gouela forest, 07°37'N, 008°21'W, Winkler extraction, 12.X.2008; 39 ex. (MRAC 21981), including a ♀ neotype of *Cenchrodesmus volutus*, same locality, Winkler extraction, 15–17.X.2008; 6 ex. (MRAC 21991), same locality, Winkler extraction, 13–15.X.2008; 4 ex. (MRAC 22004), same locality, Winkler extraction, 17–19.X.2008; 12 ex. (MRAC 22045), same locality, Winkler extraction, 13–16.X.2008; 4 ex. (MRAC 22007), Mt Nimba, Zié forest, 07°40'N, 008°22'W, Winkler extraction, 16–18.X.2008; 2 ex. (MRAC 22040), same locality, Winkler extraction, 14–16.X.2008; 2 ex. (MRAC 22050), Mt Nimba, Ziela forest, 07°43'N, 008°21'W, litter, Winkler extraction, 19.X.2008; all leg. D. VandenSpiegel; 1 ♂, 1 ♀ (MRAC 22284), Taï forest, 05°50'N, 007°21'W, Winkler extraction, 01–03.IX.2010; 1 ex. (MRAC 22285), same locality, Winkler extraction, 13–15.X.2010; 4 ex. (MRAC 22286), same locality, Winkler extraction, 01–03.IX.2010; 2 ex. (MRAC 22288), same locality, Winkler extraction, 01–03.IX.2010; 1 ex. (MRAC 22289), same locality, Winkler extraction, 01–03.IX.2010; 2 ex. (MRAC 22290), same locality, Winkler extraction, 13–15.X.2010, all leg. A. Kablan; 3 ex. (MRAC 22287), same locality, forest on clayey soil, Winkler extraction, 22–24.II.2010; 4 ex. (MRAC 22291), Taï forest, Ecological Research Centre, 05°50'N, 007°21'W, secondary forest, Winkler extraction, 21–22.II.2010, all leg. M. Diarassouba & R. Jocqué.

#### Diagnosis.

Minute polydesmidans (1.4–2.0 mm in length) showing evident sexual dimorphism in tergal structure: ♂ with a transverse row of up to ten ovoid tubercles arising from posterior part of each metatergum, ♀ with nearly smooth metaterga. Gonopod with a small globular coxa reaching in length only about one-third of telopodite; the latter slender, flattened and twisted mesad in distal part, with a small sac-shaped outgrowth laterally at base.

#### Redescription.

♂ ca 1.9 mm long; maximum width, 0.9 mm. Entire dorsal surface covered with a thin layer of secretions (= cerategument), under which the body integument is light brown to pinkish with metaterga of each segment brownish (Fig. 1). Body with 18 or 19body rings (16 or 17+1+T), shape as in Figs 1, 2 & 3, with caudal body end tapering towards a relatively small telson not concealed by paraterga (Fig. 6).

**Figures 7–15. F2:**
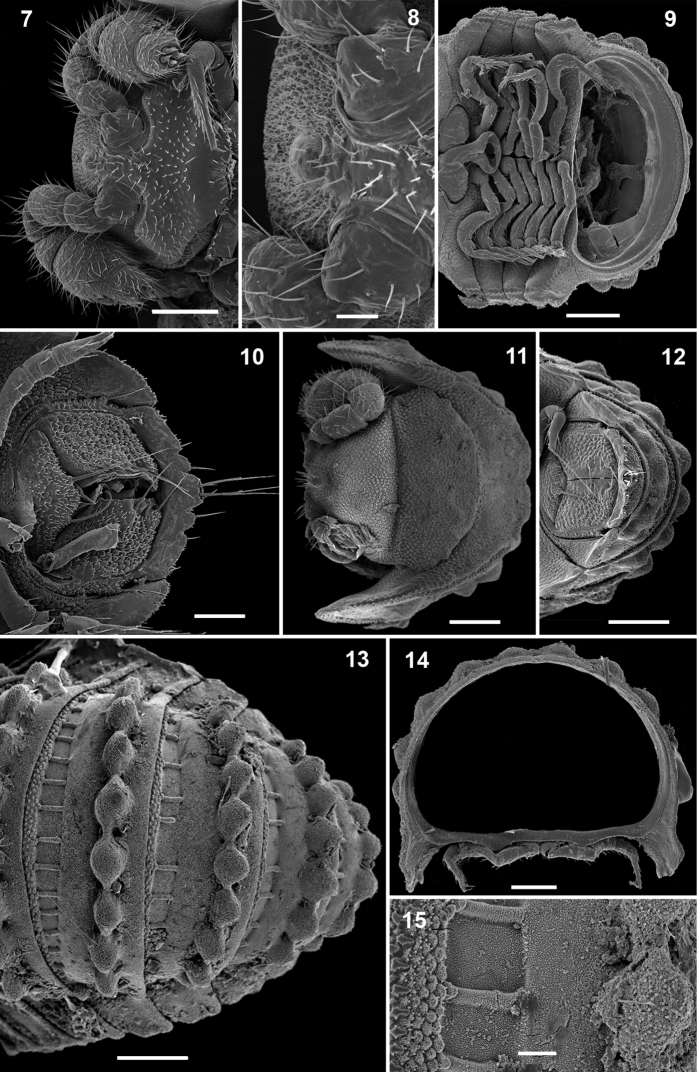
*Ammodesmus granum* **7** male head, ventral view **8** interantennal isthmus, ventral view **9** midbody segments, ventral view **10** caudal part of body, ventral view **11** head and collum, dorsal view **12, 13** posterior part of body, caudal and dorsal views, respectively **14** cross-section of a midbody segment, caudal view **15** tegument texture, dorsal view. Scale bars: **7, 9–14**, 100 µm; **8, 15**, 20 µm.

**Figures 16–23. F3:**
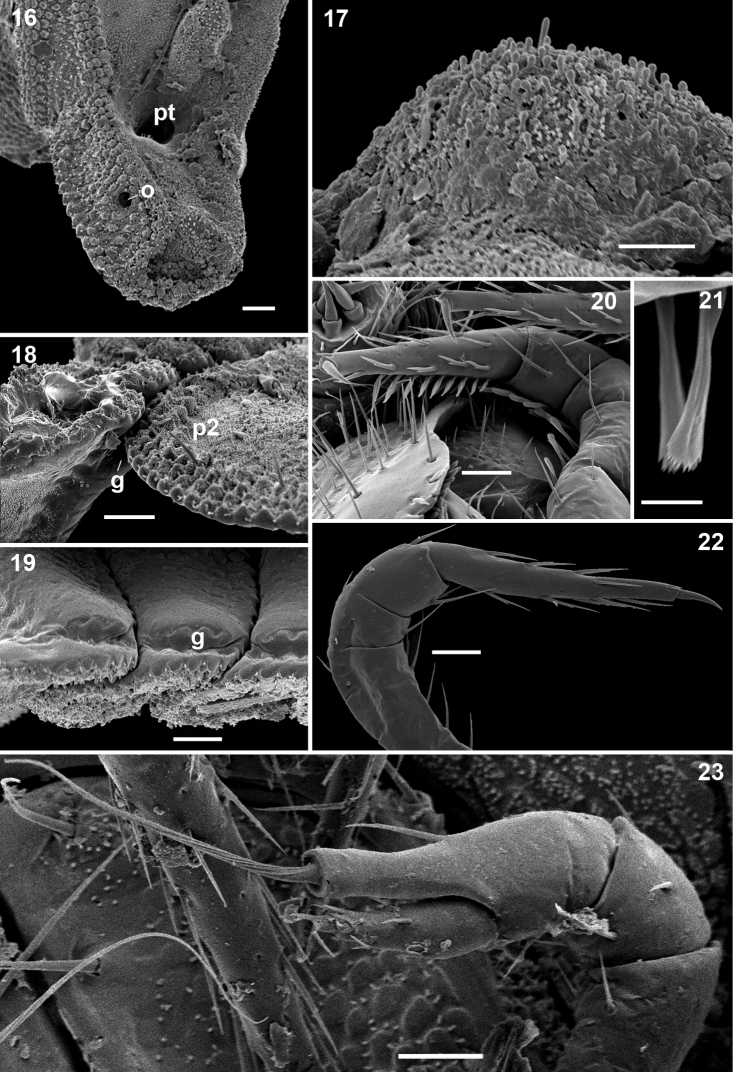
*Ammodesmus granum*, male. **16** midbody paraterga, lateral view **17** metatergal tubercle, lateral view **18, 19** paratergal groove, laterocaudal and ventral views, respectively **20** first left leg, ventral view **21** modified setae of first leg **22** midbody leg, lateral view **23** last right leg, lateral view. Scale bars: **16**, 100 µm; **17, 23**, 10 µm; **18, 19, 20, 22**, 20 µm; **21**, 5 µm (**g**: groove, **o**: ozopore, **pt**: pit, **p2**: paraterga 2).

Head small, only partly concealed under front edge of collum (Figs 7, 11); preceding half of head densely granular, lower part smooth and densely setose (Figs 7, 8). Interantennal isthmus about as wide as antennomere 1, surmounted by a small tubercle (Fig. 8). Antennae as in Fig. 7. Collum covered with low rounded tubercles (Fig. 11), tergum 2 as usual, hypertrophied, with strongly enlarged, spatuliform paraterga concealing the head in lateral view, ventral edge with a line of granules (Fig. 3). Limbus smooth; 2^nd^ and following metaterga with 7–10 large oblong tubercles along caudal margin (Figs 1, 2, 4, 5, 6, 13). Each tubercle surmounted by a short seta (Fig. 17). Prozona rugose anteriorly, with a row of square areas along anterior edge of metatergum (Figs 13, 15), these square areas being reduced in ♀ or absent in juveniles (Fig. 3). Paraterga set at segments’ midheight just below a deep pit (Fig. 16, pt), continuing the highly convex outline of dorsum, their ends rounded, projecting far below venter/coxae (Fig. 14), increasingly angular towards telson (Figs 5, 6). Anteroventral edges of paraterga 3 to 15 with a notch forming a groove for paratergum 2 to hinge into during volvation (Figs 18, 19, g). Ozopore formula: 5, 7, 9, 10, 12, 13, 15, 17; ozopores opening flush on tergal surface about midheight of paraterga, most of openings concealed by preceding paraterga (Fig. 16). Telson small (Fig. 6).

Legs rather slender, but short, barely reaching tips of paraterga (Fig. 14); femoral and tarsal segments longest, subequal in length; claw normal, simple, very slightly curved ventrad (Fig. 22); first pair of legs in ♂ with modified setae (Figs 20, 21); last pair of ♂ legs modified, typical of *Ammodesmus* (Fig. 23).

**Figures 24–27.**
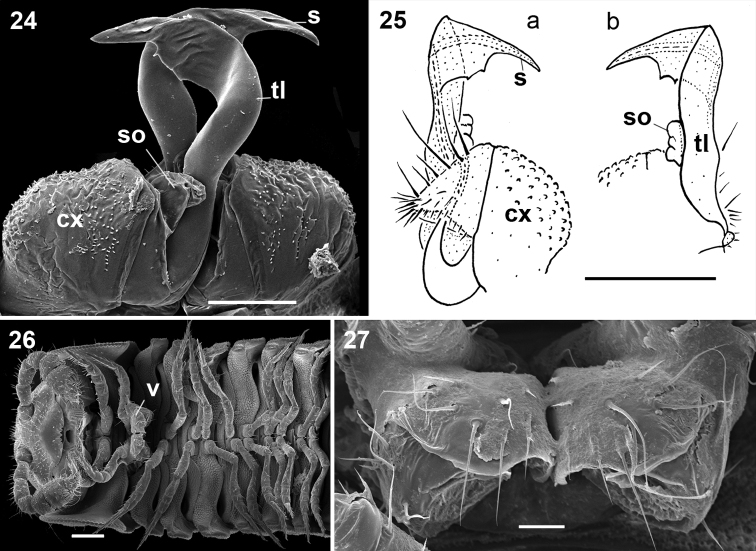
*Ammodesmus granum*. **24** gonopods, caudal view **25** drawing of right gonopod, frontal (**a**) and caudal (**b**) views, respectively **26** anterior part of female body, ventral view **27** vulvae, ventral view. Scale bars: **24**, 50 µm; **25, 26**, 100 µm; **27**, 20 µm. (**cx**: coxae, **s**: solenomere, **so**: sac-shaped outgrowth, **tl**: telopodite).

Gonopods (Figs 24, 25) relatively simple. Coxae rather small, globular, scaly, without setae. Telopodite long and well exposed beyond small gonocoxae; apical part of the main body of telopodite (= solenomere) smooth, flattened, pointed and twisted medially, devoid of a hairy pulvillus; a small, sac-shaped, lateral outgrowth at base of telopodite.

♀ usually slightly larger than ♂, segments rather smooth, without metatergal tubercles. Vulva small, poorly sclerotized, edge of bursa supplied with long setae (Figs 26, 27).

#### Distribution.

Known from Liberia, Guinea and the Ivory Coast. It is noteworthy that at Mt Nimba this species occurs parapatrically with the new congener described below.

#### Remarks.

*Ammodesmus granum* is striking in being perhaps the only species in Polydesmida in which both sexes vary in the number (18 or 19) of body rings. Infraspecific variations in the number of body segments in this order are usually quite rare, always being stable per sex. Thus, in such cases males always have fewer body rings (18 or 19) than females (19 or 20), a situation not too uncommon, e.g., in Haplodesmidae (Golovatch et al. 2009a, 2009b, 2010) and, especially, Opisotretidae (Golovatch 1988).

Neotype designations for both *Ammodesmus granum* and *Cenchrodesmus volutus* are necessary, because the original types can be presumed as being lost. A special search undertaken among Cook’s diplopod collections, currently housed in the Smithsonian Institution, National Museum of Natural History, Washington, D.C., had failed already before the description of *Elassystremma pongwe* by Hoffman and Howell (1981).

### Ammodesmus nimba sp. n.

urn:lsid:zoobank.org:act:B0262CE7-D6AC-434D-BC93-A502EAC2D999

http://species-id.net/wiki/Ammodesmus_nimba

**Figures 28–35.**
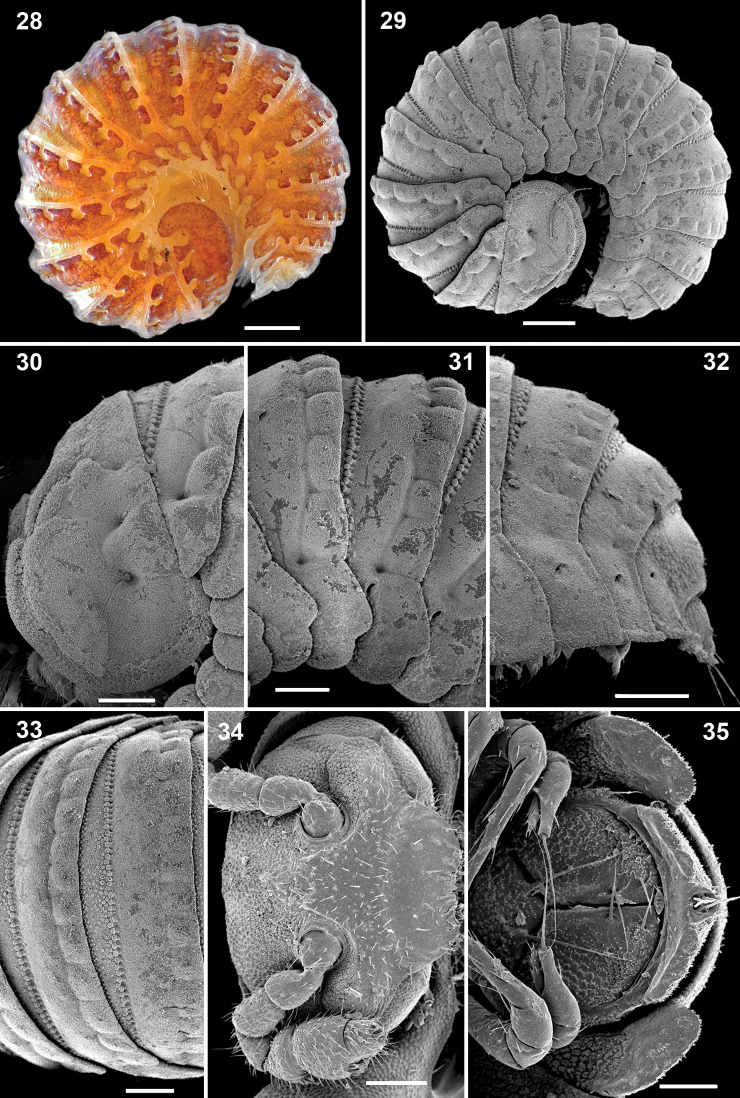
*Ammodesmus nimba* sp. n., male paratype. **28, 29** habitus, lateral view **30–32** anterior, middle and caudal parts of body, respectively, lateral view **33** midbody segments, dorsal view **34** head, frontal view **35** posterior part of body, ventral view. Scale bars: 100 µm.

Figs 28 [Fig F6] –46

#### Type material.

Holotype ♂ (MRAC 22510), GUINEA, Mt Nimba, Freton forest, 07°37'N, 008°29'W, soil and litter, Winkler extraction, 10.III.2012, leg. A. Henrard, C. Allard, P. Bimou & M. Sidibé. Paratypes: 12 ex. (MRAC 22511), 1 ♂, 1 ♀ (ZMUM), 1 ♂ (MNHN), same locality, together with holotype.

#### Diagnosis.

Minute polydesmidans with a characteristic, spotty colour pattern of the caudal edge of each segment. Gonopods with extremely large coxae concealing the telopodites inside a deep gonocoel.

#### Description.

♂ca 2.8 mm long; maximum width, 0.9 mm. Body integument light brown to pinkish. Colour pattern of metaterga characteristic, spotty (Fig. 28). Body with 19 body rings (17+1+T), shape as in Figs 28, 29.

**Figures 36–42. F6:**
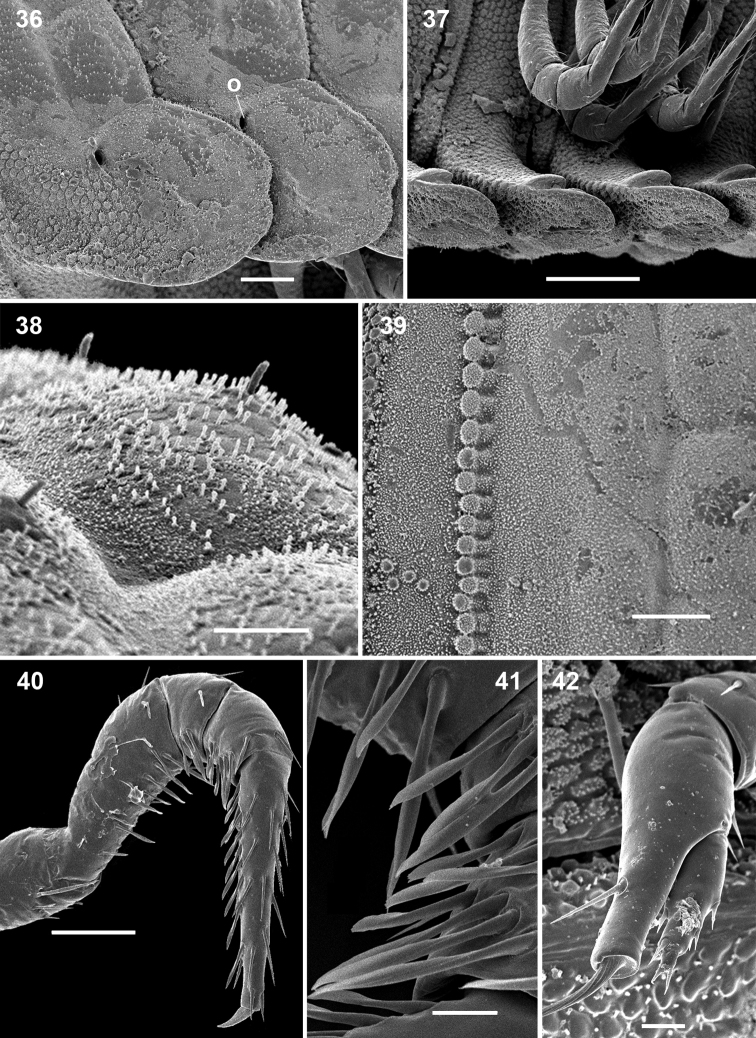
*Ammodesmus nimba* sp. n., male paratype. **36, 37** midbody paraterga, lateral and ventral views, respectively **38** metatergal bosses, sublateral view **39** tegument texture, dorsal view **40** first leg **41** modified setae of first leg **42** last right leg, lateral view. Scale bars: **36, 39, 40**, 50 µm; **37**, 100 µm; **38**, 20 µm; **41, 42**, 10 µm (**o**: ozopore).

Head small, partly concealed under front edge of collum; upper half of head densely granular, lower half smooth and densely setose. Interantennal isthmus without knob, about as wide as antennomere 1 (Fig. 34). Antennae as in Fig. 34. Collum relatively large, rather convex, surface slightly granular. Tergum 2 as usual, hypertrophied, with strongly enlarged, spatuliform paraterga concealing the head in lateral view (Figs 28, 29, 30), ventral edge with up to 4 rows of granules (Figs 29, 30). Limbus smooth, 2^nd^ and following metaterga with a row of up to 13 low bosses lining the caudal margin; each boss obviously supporting a small apical seta (Figs 30–33, 38). Prozona rugose anteriorly, with a row of small granules along anterior edge of metatergum (Fig. 39). Paraterga set below segments’ midheight, continuing the convex outline of dorsum, with a notch basally at posterolateral edge; ends rather regularly rounded, increasingly angular towards telson (Figs 31, 32, 36). Anteroventral parts of paraterga 3 to 15 with a notch forming a groove for paraterga 2 to hinge into during volvation (Fig. 37). Ozopore formula: 5, 7, 9, 10, 12, 13, 15, 17; ozopores opening flush on tergal surface at about anterior third of paraterga, openings oblong and not concealed by preceding paraterga (Fig. 36). Telson small (Figs 32, 36).

**Figures 43–46.**
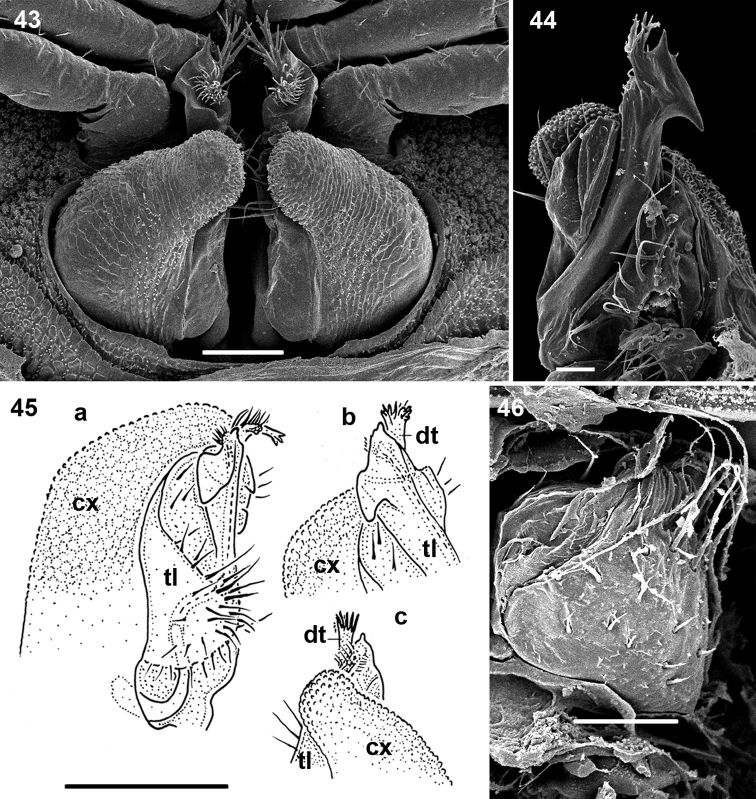
*Ammodesmus nimba* sp. n. **43** gonopods, caudal view **44** right gonopod, frontal view **45** drawing of left gonopod, frontal view (**a**), apicofrontal (**b**) and caudal (**c**) views, respectively **46** right vulva, ventral view. Scale bars: **43, 46**, 50 µm; **44**, 20 µm; **45**, 100 µm. (**cx**: coxae, **dt**: digitiform tubercle, **tl**: telopodite)

Sterna and legs as in *Ammodesmus granum* (Figs 40, 41, 42). Gonopod aperture relatively modest in size, transversely oval (Fig. 43).

Gonopods highly complex (Figs 43–45); coxae oblong, strongly enlarged to protect telopodites (Figs 43, 45). Telopodite only a little longer than coxa, showing a hook-shaped apical part (Figs 44, 45b) carrying a digitiform tubercle (Figs 43, 44, 45c). Solenomere very small and short, supplied with a distinct hairy pulvillus (Fig. 45a).

♀ agrees precisely in colour and structural details with ♂, also being (nearly) of the same size and counting 19 body rings. Vulva small, setose, poorly sclerotized, edge of bursa with some particularly long setae (Fig. 46).

#### Relationships.

**Figure 47.**
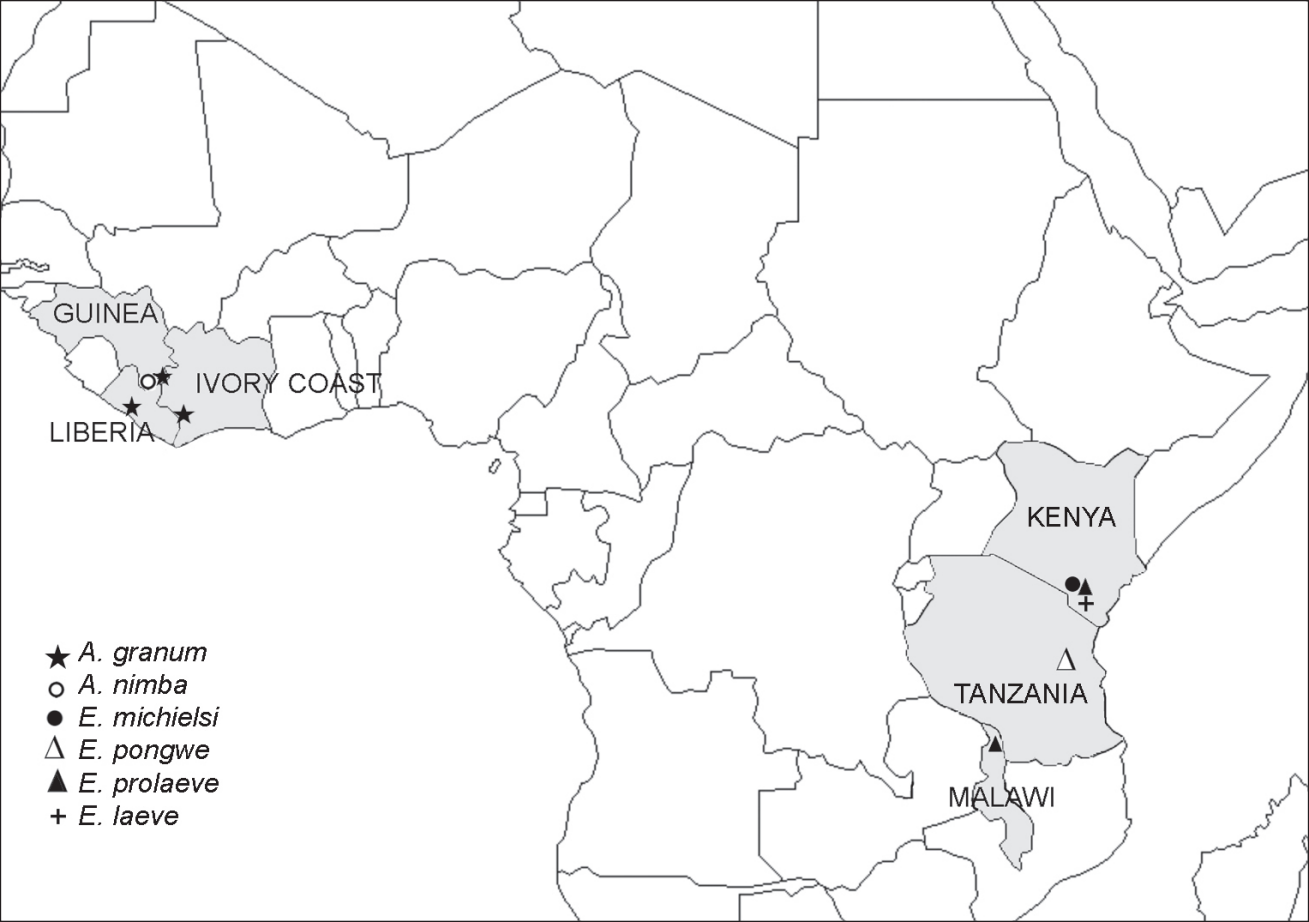
Distribution of the family Ammodesmidae.

Superficially, both species of *Ammodesmus* might look sufficiently different to consider them as representing different genera, especially as regards the absence of sexual dimorphism in metatergal structure and the presence of a deep gonocoel in *Ammodesmus nimba* as opposed to *Ammodesmus granum*. The main distinctions can also be summarized in a tabular form (Table). However, based on all evidence, we are rather inclined to recognize only two valid genera in Ammodesmidae, both quite disjunct also geographically (Fig. 47).

**Table.** Principal differences between genera of Ammodesmidae

Key characters in genera of Ammodesmidae	*Ammodesmus granum*	*Ammodesmus nimba* sp. n.	*Elassystremma* spp.
Sexual dimorphism in metatergal structure	yes	no	no
Size	up to 2 mm long	idem	up to 5 mm long
Ozopore formula	5, 7, 9, 10, 12, 13, 15–17(18)	idem	5, 7, 9, 12, 15, 17(18)
First male leg with modified setae	yes	yes	no
Last male leg modified	yes	yes	no
Gonopod telopodite deeply sunken into a very evident gonocoel	no	yes	yes

#### Name.

Referring to the type locality, a noun in apposition.

#### Distribution.

Known only from the type locality and probably endemic to Mt Nimba.

## Conclusion

Despite extensive efforts applying the same collecting techniques in similar habitats in many places, *Ammodesmus nimba* appears to occur, and to be apparently common, only at the single locality whence it has been taken, whereas *Ammodesmus granum* has a surprisingly wide distribution. The vast range of *Ammodesmus granum*, currently reported from the western part of Liberia, at Mt Nimba in Guinea and in the Taï forest in the western part of Ivory Coast, is rather unusual for such a tiny and hygrophilous animal. Certainly being likewise rather poorly vagile, this species can be assumed to represent a relict which must have been widely distributed in the past when woodlands werecontinuous in western tropical Africa (Couvreur et al. 2008).

Geographically, the family Ammodesmidae seems to be purely Afrotropical, *Ammodesmus* being obviously confined to western Africa while *Elassystremma* to eastern Africa (Fig. 47). All four *Elassystremma* species (*Elassystremma pongwe* Hoffman & Howell, 1981, *Elassystremma michielsi* VandenSpiegel & Golovatch, 2004, *Elassystremma leave* VandenSpiegel & Golovatch, 2004 and *Elassystremma prolaeve* VandenSpiegel & Golovatch, 2004) are slightly larger than *Ammodesmus* (up to 5 mm long), and their gonopods are invariably complex, sunken inside a deep gonocoel (VandenSpiegel and Golovatch 2004). Likewise, only one species, *Elassystremma prolaeve*, is widespread, occurring not only in Kenya and Malawi, but obviously also in-between in Tanzania (Fig. 47).

The use of Winkler-Mocsarski apparatuses, or Winkler apparatuses for short, appears to be the most appropriate technique in sampling particularly cryptic soil/litter fauna. This technique has allowed for material to be collected in almost any East and West African tropical forest prospected by the first author and it is most likely that new species will be revealed in the central parts of the continent, if the collecting efforts use appropriate techniques. At least the wide geographical gap between both genera certainly invites further studies (Hoffman 1993). More refinements to the distribution of already known species are also most plausible.

## Supplementary Material

XML Treatment for Ammodesmidae

XML Treatment for Ammodesmus

XML Treatment for Ammodesmus granum

XML Treatment for Ammodesmus nimba

## References

[B1] Cook OF (1895) Introductory note on the families of Diplopoda. - In: Cook OF Collins GN (Eds) , The Craspedosomatidae of North America.Annals of the New York Academy of Sciences 9: 1–100

[B2] Cook OF (1896a) East African Diplopoda of the suborder Polydesmoidea, collected by Mr. William Astor Chanler. Proceedings of the United States National Museum 18: 81–111 [for 1895].

[B3] Cook OF (1896b) A new diplopod fauna in Liberia. The American Naturalist 30: 413-420

[B4] Cook OF (1896c) Summary of new Liberian Polydesmoidea. Proceedings of the Academy of Natural Sciences ofPhiladelphia 1896: 257-267

[B5] Couvreur TLP Chatrou LW Sosef MSM Richardson JE (2008) Molecular phylogenetics reveal multiple tertiary vicariance origins of the African rain forest trees. BMC Biology 2008, 6: 1–10. 10.1186/1741-7007-6-54 PMC2628871 19087283

[B6] Golovatch SI (1988) On the first Polydesmidae, Opisotretidae and Fuhrmannodesmidae from Bhutan (Diplopoda, Polydesmida). Entomologia Basiliensia 12: 15-48

[B7] Golovatch SI (2003) A review of the volvatory Polydesmida, with special reference to the patterns of volvation (Diplopoda). African Invertebrates 44 (1): 39-60

[B8] Golovatch SI Geoffroy JJ Mauriès JP VandenSpiegel D (2009a) Review of the millipede family Haplodesmidae (Diplopoda: Polydesmida), with descriptions of some new or poorly-known species. ZooKeys 7: 1-53

[B9] Golovatch SI Geoffroy JJ Mauriès JP VandenSpiegel D (2009b) Review of the millipede genus *Eutrichodesmus* Silvestri, 1910 (Diplopoda: Polydesmida: Haplodesmidae), with descriptions of new species. ZooKeys 12: 1-46 10.3897/zookeys.505.9862 PMC4453233 26052236

[B10] Golovatch SI Mikhaljova EV Korsós Z Chang HW (2010) The millipede family Haplodesmidae (Diplopoda, Polydesmida) recorded in Taiwan for the first time, with the description of a new species. Tropical Natural History 10 (1): 27-36

[B11] Hoffman RL (1980) Classification of the Diplopoda. Muséum d’histoire naturelle, Genève, 237 pp. [for 1979]

[B12] Hoffman RL (1993) Biogeography of East African montane forest millipedes. - In: Lovett JC, Wasser SK (Eds), Biogeography and ecology of the rain forests of eastern Africa: 103–114.

[B13] Hoffman RL Howell KM (1981) An ammodesmid millipede from Tanzania (Polydesmida). Revue de Zoologie africaine 95 (1): 227-233

[B14] Jeekel CAW (1970) Nomenclator generum et familiarum Diplopodorum: A list of the genus and family-group names in the Class Diplopoda from the 19^th^ edition of Linnaeus, 1758, to the end of 1957. Monografieën van de Nederlandse Entomologische Vereniging 5: i-xii + 1–412.

[B15] VandenSpiegel D Golovatch SI (2004) Review of the East African millipede genus *Elassystremma* Hoffman & Howell, 1981 (Diplopoda: Polydesmida: Ammodesmidae), with descriptions of three new species. Arthropoda Selecta 12(3–4): 183–191 [for 2003].

